# Optimal Design of Shape Memory Alloy Composite under Deflection Constraint

**DOI:** 10.3390/ma12111733

**Published:** 2019-05-28

**Authors:** Yogesh Gandhi, Alessandro Pirondi, Luca Collini

**Affiliations:** Department of Engineering and Architecture, University of Parma, Parco Area delle Scienze, 181/A, 43124 Parma, Italy; alessandro.pirondi@unipr.it (A.P.); luca.collini@unipr.it (L.C.)

**Keywords:** smart composite, numerical optimization, shape memory alloy, snap-through, bi-stability

## Abstract

Shape-adaptive or morphing capability in both aerospace structures and wind turbine blade design is regarded as significant to increase aerodynamic performance and simplify mechanisms by reducing the number of moving parts. The underlying bistable behavior of asymmetric cross-ply composites makes them a suitable candidate for morphing applications. To date, various theoretical and experiential studies have been carried out to understand and predict the bistable behavior of asymmetric laminates and especially the curvature obtained in their stable configurations. However, when the bi-stable composite plate is integrated with shape memory alloy wires to control the curvature and to snap from a stable configuration to the other (shape memory alloy composite, SMAC), the identification of the design parameters, namely laminate edge length, ply thickness and ply orientation, is not straightforward. The aim of this article is to present the formulation of an optimization problem for the parameters of an asymmetric composite laminate integrated with pre-stressed shape memory alloys (SMA) wires under bi-stability and a minimum deflection requirement. Wires are modeled as an additional ply placed at the mid-plane of the composite host plate. The optimization problem is solved numerically in MATLAB and optimal design variables are then used to model the SMAC in ABAQUS™. Finite element results are compared against numerical results for validation.

## 1. Introduction

Morphing structures applications are of interest in both aerospace structures [1] and wind turbines [2], which take benefit of significant shape changes to increase performance and efficiency. Asymmetric composite laminates (ACL) may be formulated such that they possess two stable shapes, each of which is a natural equilibrium position. Therefore, ACL can settle at either of them without the need of an external aid. The bi-stable behavior of ACL is related to the residual stress field that develops after curing due to a mismatch in coefficient of thermal expansion in an unsymmetric stacking sequence. The application of bending and twisting moments with respect to mid-plane, can result in a new internal stress equilibrium at a second stable configuration. The state change can be obtained by inducing in-plane strains, typically by smart actuators such as piezoelectric and shape memory alloys (SMA). The intrinsic anisotropy of composite structures was first exploited by Hyer [3,4] using unsymmetric layups to produce bi-stable morphing structure at room temperature. Jun and Hong [5] modified Hyer’s theory by taking into account in-plane shear strain. Schlecht and Schulte [6] firstly provided the bi-stable behavior of asymmetric laminates using MARC Finite Element Analysis (FEA) software. Tawfik et al. [7] presented a finite element approach using ABAQUS™ to predict the unsymmetric laminate shapes under thermal curing stresses.

Shape memory alloys have functional properties associated with the pseudo-elastic behavior and shape memory effect (SME). The pseudoelasticity effect allows the alloy to deform elastically up to 7–8% strain, that means about forty times the elastic regime of most steels. Shape-memory alloys can also remember their original shape (SME), after being pseudoplastically deformed below a given temperature (transformation temperature), just deformed by increasing their temperature. The SME is due to the thermoelastic austenite-martensite transformation, which can be described as a diffusion-less process. One of the major advantages of SMAs over other actuator materials is their large recovery force per unit area generated by the phase transformation following the initial deformation. In addition, due to its high tensile strength, SMAs are appropriate for use in small-sized, high-output actuators. Several SMA constitutive models have been proposed and developed over the past few decades to describe the SMA phase transformation phenomenon [8,9,10,11,12,13,14]. However, SMA based actuators [15] have a slow actuation speed which restricts their use in most of the nanotechnology applications such as RF filters, fluid sensors, or devices for the quantum state measurement. Stachiv et al. [16] design tunable high frequency SMA microcantilevers resonators and demonstrated that the phase changeable SMA film can be used by enabling tunability of several consecutive resonant frequencies. Here, in contrast to the usual thermally actuated SMA, the high frequency actuation is generated by the elastic substrate material. Furthermore, Stachiv et al. [17] also proposed nanocantilevers with a tunable spectrum of the resonant frequencies and changeable static deflection utilizing the phase transformation of NiTi film sputtered on the elastic substrate material. 

An SMA-embedded composite could be used as an integrated smart actuator–structure without any additional or separated actuation device. As a result, it offers a significant contribution to the design of lightweight structures. Ryu et al. [18] verified actuation of asymmetric laminate using SMA spring actuator through the comparison between experiment and numerical simulation. Dano and Hyer [19] used a mechanism wherein, after SMA wires were stretched between a system of support placed above the laminate and upon electrical heating of the SMA wires, it was concluded that is possible to use SMA wires to change the shape of unsymmetric laminates and to predict reasonably well the overall response of the laminate as a function of the SMA wire temperature. Hassanli and Samali [20] investigated the buckling of curved laminated composite panels reinforced with SMA fibers. A different finite element method was used by Tawfik et al. [21] to examine the stability behavior of SMA composite panels. In addition, Von Karman nonlinear strains were considered in the formulations. Turner [22] examined the thermoelastic response of SMA hybrid structures by finite element analysis. Niknami et al. [23] investigated the effect of induced heat generations on impact responses and phase transformations of hyrbid SMA composite plate through proposing a refined Helmholtz free energy expression and refined constitutive and contact laws, in addition to employing a return-map Newton-Raphson method for enhancement of the numerical solution algorithm. Birman et al. [24] illustrate that SMA fibres embedded within the layers of a composite plate can significantly enhance its global resistance to low-velocity impact and the effectivness of SMA fibre can be further improved by optimizing their distribution throughout the plate. Gandhi et al. [25] presented the possibility to trigger the snap-through from one stable configuration to another by means of SMA wires embedded into the laminate. 

Moving on from the work done in [25], the aim of the present study is to predict the optimal design variables for an asymmetric, square laminate with embedded SMA wires such that the laminate can attain a desired stable configuration after the cure cycle and a second stable configuration is admissible, that can be attained by snap from the first one actuated with the help of SMA wires. However, the need of a detailed investigation to identify the optimal design variables necessary to obtain the actuation, requires the definition of an optimization technique in order to avoid a time-consuming trial-and-error procedure, that is based on intuition with no prior knowledge about the obtained configuration and out-of-plane displacement. While a square, asymmetric laminate was selected based on the fact it allows a bistable behavior and a theoretical framework for optimization without SMA is already present in the literature [26], it is acknowledged that this will not cover all laminate configurations which may be considered for practical applications as, for example, rectangular laminates. The optimization study considers the design of bi-stable laminates through variation in ply orientations, ply thickness (same for all plies), and laminate edge length. An objective function has been defined to optimize the ratio of bending stiffness in two chosen direction of the laminate in order to control the shape of the laminate after the curing process. The search space comprises of multiple local and global optima that vary with the bounds posed on the design variables and the deflection constraint. MATLAB’s sequential quadratic programming method [27,28] is used to solve the optimization problem. Given the complex and multimodal nature of the laminate design problem, MultiStart Algorithm is used to generate random points within the bounds to capture all local optima and search thoroughly for the global minimum. The optimization problem is solved, as an example, for [45°, 0] and [75°, 0°] low and high stiffness bending direction to find optimal geometric design variable when the laminate is subjected to deflections and bi-stability constraints. The accuracy of the optimal design is verified against SMAC model implemented in ABAQUS to predict the shape after curing and the response of the model.

## 2. Governing Equations

### 2.1. Constitutive Model of SMA Wires

The constitutive model for SMA response to mechanical and thermal load is available as a user-defined material model (UMAT) for the ABAQUS™ 6.13 software [29], that is a FORTRAN (Formula Translation) code numerical implementation compiled against the Intel Fortran compiler v13.1 and linked into an ABAQUS executable via Microsoft Visual Studio 2012 Professional (Microsoft, Redmond, WA, USA).

This particular constitutive model is rigorously developed to ensure that the key constitutive relations and evolution equations are thermodynamically sound. A detailed discussion of the model derivation process can be found in the works of Lagoudas et al. [13], and so will not be covered in this work. However, a brief summary of the resulting constitutive relations and transformation conditions is outlined here.

The total strain tensor ε is additively decomposed into elastic strain $\varepsilon^{el}$, thermoelastic strain $\varepsilon^{th}$, and transformation strain $\epsilon^{t}$:

(1) $$\varepsilon =\varepsilon^{el}+\varepsilon^{th}+\epsilon^{t},$$

For simplicity, this work assumes that the only inelastic strain component is the transformation strain $\epsilon^{t}$ and does not take in account for the evolution of transformation-induced plastic strain in SMA or consider the reorientation of variants of martensite.

The constitutive equation is stated as:

(2) $$\varepsilon =S\left(\xi \right)\sigma +\alpha \left(T-T_{0}\right)+\epsilon^{t},$$

where ***α*** is a second-order thermal expansion coefficient tensor and S is a fourth-order compliance tensor. The compliance tensor is a function of the evolving martensite volume fraction *ξ* and the compliance tensors $S^{A}$ and $S^{M}$ for the austenite and martensite phases, respectively, and is formulated as a rule of mixtures given by:

(3) $$S\left(\xi \right)=S^{A}+\xi (S^{M}-S^{A}),$$

The evolution equation, which is responsible for relating the time rate of change of transformation strain with the time rate of change of the internal state variable *ξ*, assumes the form of a flow rule:

(4) $$\dot{\epsilon^{t}}=\Lambda \dot{\xi },$$

Here, the transformation tensor Λ indicates the direction of transformation with the branching function:

(5) $$\Lambda =\{\begin{array}{l} \frac{3}{2}H^{max}\frac{\sigma^{\prime }}{{\bar{\sigma }}^{\prime }}\;;\;\dot{\xi }>0\\ H^{max}\frac{\epsilon^{t-r}}{{\bar{\epsilon }}^{t-r}}\;;\;\;\dot{\xi }<0 \end{array},$$

where $H^{max}$ is a material parameter associated with the maximum transformation strain, and $\epsilon^{t-r}$ and ${\bar{\epsilon }}^{t-r}$ are the transformation strain and the effective transformation strain at the point of transformation reversal (i.e., the point at which the material stops forward transforming and begins reverse transforming). For reference, the deviatoric stress $\sigma^{\prime }$ is given by:

(6) $$\sigma^{\prime }=\sigma -\frac{1}{3}tr\left(\sigma \right)I,$$

while the associated effective von Mises scalar measure of the stress tensor ${\bar{\sigma }}^{\prime }$ is defined by:

(7) $${\bar{\sigma }}^{\prime }=\sqrt{\frac{3}{2}{\left\|\sigma^{\prime }\right\|}^{2}},$$

The effective transformation strain at the reversal of the phase transformation is given by:

(8) $${\bar{\epsilon }}^{t-r}=\sqrt{\frac{2}{3}\left\|\epsilon^{t-r}\right\|{}^{2}},$$

The phase transformation of the SMA is described by a transformation function, Φ=Φ(σ,T,ξ), such that:

(9) $$\Phi =\{\begin{array}{c} \pi -Y,\;\;\dot{\xi }>0\\ -\pi -Y,\;\;\dot{\xi }<0 \end{array},$$

where *Y* is the critical thermodynamic driving force necessary to initiate transformation, the current value (*π*) of which is given by:

(10) $$\pi \left(\sigma ,T,\xi \right)=\sigma :\Lambda +\frac{1}{2}\sigma :\Delta S:\sigma +\sigma :\Delta \alpha \left(T-T_{0}\right)-\rho \Delta c\left(\left(T-T_{0}\right)-T\;ln\left(\frac{T}{T_{0}}\right)\right)\;+\rho \Delta s_{0}T-\rho \Delta u_{0}-\frac{\partial f}{\partial \xi },$$

where Δ(**·**) refers to difference in the material properties between the martensite and austenite phases, i.e.; Δ(**·**) = (**·**)^M^ − (**·**)^A^, ρ is the material density, c is the specific heat capacity, $s_{0}$ and $u_{0}$ are the respective reference entropy and internal energies, and f(ξ) is a chosen transformation hardening function. As seen in Equation (10), transformation occurs when π value equals Y. Thus, during transformation Φ is necessarily zero. From the above relations, the evolution of the martensitic volume fraction is said to be governed by the set of constraints called the Kuhn-Tucker conditions. These conditions are concisely stated as:

(11) $$\dot{\xi }\geq 0;\;\Phi \left(\sigma ,T,\xi \right)=\pi -Y\leq 0;\;\Phi \dot{\xi }=0,\;\dot{\xi }\leq 0;\;\Phi \left(\sigma ,T,\xi \right)=-\pi -Y\leq 0;\;\Phi \dot{\xi }=0,$$

With the equations described here, the constitutive model is implemented in the finite element framework.

### 2.2. The Constitutive Model of SMAC

#### 2.2.1. Mathematical Modelling for Composite Laminate

The model to predict the cured shape is based on a nonlinear extension of the classical laminated plate theory with approximated midplane strain functions and nonzero in-plane shear strain. The coordinate system used is such that origin is placed at the geometric center of the laminate and plies are defined in order starting from the bottom (negative thickness coordinate) surface. The midplane strains including geometrical nonlinearity according to the von Karman hypothesis are defined as:

(12) $$\left\{\begin{array}{c} \varepsilon^{0}{}_{x}\\ \varepsilon^{0}{}_{y}\\ \gamma^{0}{}_{xy} \end{array}\right\}=\left[\begin{array}{c} \frac{\partial u_{0}}{\partial x}+\frac{1}{2}{\left(\frac{\partial w_{0}}{\partial x}\right)}^{2}\\ \frac{\partial v_{0}}{\partial y}+\frac{1}{2}{\left(\frac{\partial w_{0}}{\partial y}\right)}^{2}\\ \frac{\partial u_{0}}{\partial y}+\frac{\partial v_{0}}{\partial x}+\frac{\partial w_{0}}{\partial x}\frac{\partial w_{0}}{\partial y} \end{array}\right],$$

where $u_{0}$, $v_{0}$ and $w_{0}$ are the in-plane displacements in the x-, y- and z-directions respectively. Therefore, the total strains distribution is given by the Kirchhoff hypothesis as:

(13) $$\left\{\begin{array}{c} \varepsilon_{x}\\ \varepsilon_{y}\\ \gamma_{xy} \end{array}\right\}=\left\{\begin{array}{c} \varepsilon^{0}{}_{x}\\ \varepsilon^{0}{}_{y}\\ \gamma^{0}{}_{xy} \end{array}\right\}+z\left\{\begin{array}{c} \kappa^{0}{}_{x}\\ \kappa^{0}{}_{y}\\ \kappa^{0}{}_{xy} \end{array}\right\};\left[\begin{array}{c} \kappa_{x}=-\frac{\partial^{2}w_{0}}{\partial x^{2}}\\ \kappa_{y}=-\frac{\partial^{2}w_{0}}{\partial y^{2}}\\ \kappa_{xy}=-2\frac{\partial^{2}w_{0}}{\partial x\partial y} \end{array}\right],$$

where $\varepsilon^{0}{}_{xx}$, $\varepsilon^{0}{}_{yy},\;\gamma^{0}{}_{xy}$ and $\kappa^{0}{}_{x}$, $\kappa^{0}{}_{y}$, $\kappa^{0}{}_{xy}$ are the total midplane strains and curvature respectively.

Since the composite laminate is subjected to the thermal load, the constitutive law of off-axis stress-strain relation in the macro-mechanical behavior of a lamina can be presented as stress-strain relations of the kth layer in a multilayered laminate.

(14) $$\left\{\begin{array}{c} \sigma_{x}\\ \sigma_{y}\\ \tau_{xy} \end{array}\right\}={\left[\begin{array}{ccc} {\bar{Q}}_{11} & {\bar{Q}}_{12} & {\bar{Q}}_{16}\\ {\bar{Q}}_{12} & {\bar{Q}}_{22} & {\bar{Q}}_{26}\\ {\bar{Q}}_{16} & {\bar{Q}}_{26} & {\bar{Q}}_{66} \end{array}\right]}_{k}\left\{\left\{\begin{array}{c} \varepsilon_{x}\\ \varepsilon_{y}\\ \gamma_{xy} \end{array}\right\}-\left\{\begin{array}{c} \alpha_{x}\\ \alpha_{y}\\ 2\alpha_{xy} \end{array}\right\}\Delta T\right\},$$

(15) $$\left\{\begin{array}{c} \varepsilon^{Tot}{}_{x}\\ \varepsilon^{Tot}{}_{y}\\ \gamma^{Tot}{}_{xy} \end{array}\right\}=\left\{\begin{array}{c} \varepsilon^{0}{}_{x}\\ \varepsilon^{0}{}_{y}\\ \gamma^{0}{}_{xy} \end{array}\right\}-\left\{\begin{array}{c} \alpha_{x}\\ \alpha_{y}\\ 2\alpha_{xy} \end{array}\right\}\Delta T,$$

where α’s are the thermal expansion coefficients in the x-y plane and are defined as:

(16) $$\left\{\begin{array}{c} \alpha_{x}\\ \alpha_{y}\\ \alpha_{xy} \end{array}\right\}=\left[\begin{array}{ccc} \cos^{2}\theta & \sin^{2}\theta & -2\cos\theta \sin\theta \\ \sin^{2}\theta & \cos^{2}\theta & 2\cos\theta \sin\theta \\ \cos\theta \sin\theta & -\cos\theta \sin\theta & \cos^{2}\theta -\sin^{2}\theta \end{array}\right]\left\{\begin{array}{c} \alpha_{1}\\ \alpha_{2}\\ 0 \end{array}\right\},$$

where $\alpha_{1}$ and $\alpha_{2}$ are the material thermal expansion coefficients in the fiber and transverse directions respectively and ΔT denotes the change in temperature from cure to operating temperature. The transformation stiffness ${\bar{Q}}_{ij}$ and the transformation matrix presented in the following matrix form:

(17) $${\left[\bar{Q}\right]}_{k}=\left[R\right]{\left[Q\right]}_{k}{\left[R\right]}^{T};\;\left[R\right]=\left[\begin{array}{ccc} \cos^{2}\theta & \sin^{2}\theta & -2\cos\theta \sin\theta \\ \sin^{2}\theta & \cos^{2}\theta & 2\cos\theta \sin\theta \\ \cos\theta \sin\theta & -\cos\theta \sin\theta & \cos^{2}\theta -\sin^{2}\theta \end{array}\right],$$

${\left[Q\right]}_{k}$ is the reduced stiffness matrix of the laminate defined as follows:

(18) $$\left[Q\right]=\left[\begin{array}{ccc} Q_{11} & Q_{12} & 0\\ Q_{12} & Q_{22} & 0\\ 0 & 0 & Q_{66} \end{array}\right];\;\left[\begin{array}{c} Q_{11}=\frac{E_{11}}{1-\upsilon_{12}\upsilon_{21}}\\ Q_{22}=\frac{E_{22}}{1-\upsilon_{12}\upsilon_{21}}\\ Q_{12}=\frac{E_{11}\upsilon_{21}}{1-\upsilon_{12}\upsilon_{21}}\\ Q_{66}=G_{12} \end{array}\right],$$

where $E_{11}$ is the longitudinal Young’s modulus, $E_{22}$ is the transverse Young’s modulus, $G_{12}$ is the shear modulus, $\upsilon_{12}$ is the major Poisson’s ratio, and the minor Poisson’s ratio $\upsilon_{21}$. The respective values used in this work are shown in Table 1. 

The resultant forces and moments acting on a laminate are obtained by integration of the stresses in each lamina through the laminate thickness. The entire collection of force and moment resultants for an *N* layered laminate for mechanical and thermal strain are defined as,

(19) $$\left\{\begin{array}{c} N_{x}\\ N_{y}\\ N_{xy} \end{array}\right\}=\int_{-H/2}^{H/2}{\left\{\begin{array}{c} \sigma_{x}\\ \sigma_{y}\\ \tau_{xy} \end{array}\right\}}_{k}dz=\overset{N}{\underset{k=1}{\sum }}\int_{-H/2}^{H/2}\left\{\begin{array}{c} \sigma_{x}\\ \sigma_{y}\\ \tau_{xy} \end{array}\right\}dz$$

(20) $$\left\{\begin{array}{c} M_{x}\\ M_{y}\\ M_{xy} \end{array}\right\}=\int_{-H/2}^{H/2}{\left\{\begin{array}{c} \sigma_{x}\\ \sigma_{y}\\ \tau_{xy} \end{array}\right\}}_{k}zdz=\overset{N}{\underset{k=1}{\sum }}\int_{-H/2}^{H/2}\left\{\begin{array}{c} \sigma_{x}\\ \sigma_{y}\\ \tau_{xy} \end{array}\right\}zdz.$$

Since the middle surface strains and curvatures are not a function of z (because these values are always at the middle surface z = 0), they need not be included in the integration. Also, the laminate stiffness matrix is constant for a given ply so it will be a constant over the integration of a lamina thickness, too. Substituting the stress-strain relation of Equation (14) and pulling these constants to the front of the integral, we obtain the following constitutive relation for laminate in matrix notation:

(21) $$N=A\varepsilon^{Tot}+B\kappa ,\;M=B\varepsilon^{Tot}+D\kappa$$

where,

(22) $$A_{ij}=\sum_{k=1}^{N}{\bar{Q}}_{{ij}_{k}}\left(z_{k}-z_{k-1}\right),B_{ij}=\frac{1}{2}\sum_{k=1}^{N}{\bar{Q}}_{{ij}_{k}}\left(z^{2}{}_{k}-z^{2}{}_{k-1}\right)\;\mathrm{and}\;\;D_{ij}=\frac{1}{3}\sum_{k=1}^{N}{\bar{Q}}_{{ij}_{k}}\left(z^{3}{}_{k}-z^{3}{}_{k-1}\right).$$

The $A_{ij}$ are extensional stiffnesses, the $B_{ij}$ are bending-extension coupling stiffnesses, and the $D_{ij}$ are bending stiffnesses. The presence of the *B_ij_* implies coupling between bending and extension of a laminate, because both forces and curvatures as well as moments and strains simultaneously exist.

#### 2.2.2. Integration of SMA Wire in Composite Laminate

**Figure 1:**
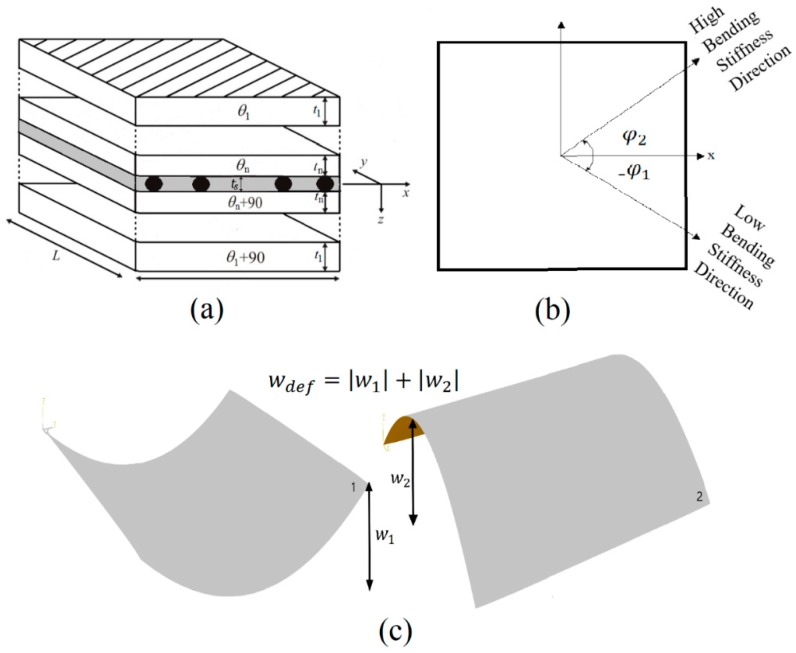
(**a**) Schematic of n-ply laminate (white) geometry with SMA/Epoxy layer (grey); (**b**) Directional stiffness positive in anticlockwise direction; (**c**) Deflection between two stable states for a cross-ply laminate.

The additional SMA layer is accommodated at the mid-plane of the composite host plate, assuming that epoxy resin fills in the space within wires such that even and an equal number of composite plies are placed on the top and bottom surface of epoxy/SMA layer as shown in Figure 1a. This approach requires a volume fraction method to combine the properties of the SMA and epoxy taken from [24]. Accordingly, the thermo-elastic properties of an epoxy/SMA layer are written as

(23) $$\left[\begin{array}{c} E^{s}{}_{11}=E_{m}V_{m}+E_{s}V_{s}\\ E^{s}{}_{22}=E_{s}E_{m}/(E_{m}V_{s}+E_{s}V_{m})\\ \upsilon^{s}{}_{12}=\upsilon_{m}V_{m}+\upsilon_{s}V_{s}\\ G^{s}{}_{12}=G_{s}G_{m}/(G_{m}V_{s}+G_{s}V_{m})\\ \alpha^{s}{}_{11}=\alpha_{m}V_{m}+\alpha_{s}V_{s}\\ \alpha^{s}{}_{22}=\alpha_{s}\alpha_{m}/(\alpha_{m}V_{s}+\alpha_{s}V_{m})\\ \upsilon_{21}=\upsilon_{12}\frac{E^{s}{}_{22}}{E^{s}{}_{11}} \end{array}\right],$$

where the “*m*” and “*s*” subscripts stand for the composite matrix and SMA fibers, respectively, and the value of $V_{s}$ is

(24) $$V_{s}=n\frac{\pi d_{s}^{2}}{Lt_{s}}$$

where n, $d_{s}$ and $t_{s}$ stands for number of SMA wires, the diameter of SMA wire and epoxy/SMA ply thickness respectively. Material parameters $E^{s}{}_{11}$, $E^{s}{}_{22}$, $G^{s}{}_{12}$, $\upsilon^{s}{}_{12}$, $\alpha^{s}{}_{11}$, $\alpha^{s}{}_{22}$, ${\bar{Q^{s}}}_{ij}$, denote therefore the values of the corresponding SMA/epoxy ply. The homogenization of SMA and epoxy properties across a ply according to Equations (23) and (24) has been chosen as a simple way to introduce SMA wires contribution in the plate. Of course, it is recognized that, when a small number of wires is considered, this is a simplifying assumption since the layer containing SMA wires is strongly heterogenous. The material parameters of the epoxy/SMA ply are evaluated using the values reported in Table 2 and Table 3. The stress influence coefficients values as shown in Table 2 has been modified to keep the consistency of sign in the article. However, for FEA simulation these coefficients must be modified back to its original form of $\rho \Delta s^{A}$ and $\rho \Delta s^{M}$ which can be achieved as follows

(25) $$\begin{array}{c} C^{A}=-\frac{\rho \Delta s^{A}}{H}\\ C^{M}=-\frac{\rho \Delta s^{M}}{H} \end{array}.$$

Since there is a large change in Young’s modulus of SMA with phase transformation, which is reflected in Equation (23), leads to established a relation between the SMAC stiffness matrix and ξ. However, for the number of wires that can be embedded in practice, as considered in this paper, the value of $V_{s}$ makes the contribution of SMA wires to the stiffness of the laminate negligible. Based on this, it is possible to assume that the change in the $E_{s}$ due to phase transformation can be neglected and $E_{s}$ can be set to a constant value equal to that at the end of the transformation, simplifying the optimization problem. 

The constitutive equations of off-axis stress-strain relation for epoxy/SMA ply with arbitrary wire orientation can be presented as follows [23]:

(26) $$\left\{\begin{array}{c} \sigma^{s}{}_{x}\\ \sigma^{s}{}_{y}\\ \tau^{s}{}_{xy} \end{array}\right\}=\left[\begin{array}{ccc} {\bar{Q^{s}}}_{11} & {\bar{Q^{s}}}_{12} & {\bar{Q^{s}}}_{16}\\ {\bar{Q^{s}}}_{12} & {\bar{Q^{s}}}_{22} & {\bar{Q^{s}}}_{26}\\ {\bar{Q^{s}}}_{16} & {\bar{Q^{s}}}_{26} & {\bar{Q^{s}}}_{66} \end{array}\right]\left\{\left\{\begin{array}{c} \varepsilon_{xx}\\ \varepsilon_{yy}\\ \gamma_{xy} \end{array}\right\}-\left\{\begin{array}{c} \alpha^{s}{}_{xx}\\ \alpha^{s}{}_{yy}\\ 2\alpha^{s}{}_{xy} \end{array}\right\}\Delta T\right\}-\left\{\begin{array}{c} V_{s}E_{s}\xi \epsilon^{t}\;\cos^{2}\theta \\ V_{s}E_{s}\xi \epsilon^{t}\;\sin^{2}\theta \\ V_{s}E_{s}\xi \epsilon^{t}\;\cos\theta \;\sin\theta \end{array}\right\}.$$

The appearance of the Martensite Volume Fraction (MVF), ξ, and the Transformation Strain (TRNS), $\epsilon^{t}$, in the stress-strain relation for the epoxy/SMA ply in Equation (26) manifest the presence of the SMA wires on the resultant force developed inside the epoxy/SMA ply. Hence, the total forces per unit length of the SMAC can be expressed as:

(27) $$\left\{\begin{array}{c} N^{T}{}_{x}\\ N^{T}{}_{y}\\ N^{T}{}_{xy} \end{array}\right\}=\left\{\begin{array}{c} N_{x}\\ N_{y}\\ N_{xy} \end{array}\right\}-\left\{\begin{array}{c} N^{s}{}_{x}\\ N^{s}{}_{y}\\ N^{s}{}_{xy} \end{array}\right\},$$

where, $N^{s}{}_{x}$ stands for resultant force in the epoxy/SMA ply which can be calculated by substituting Equation (26) into Equation (19). The linear superposition principle can be used to calculate the total internal force based on the taken assumption that stiffness computed for epoxy/SMAC is constant. Hence, the net force caused by laminate and epoxy/SMA ply is the sum of the responses that would have been caused by each stimulus individually.

### 2.3. Optimization of SMAC

Betts [26] presented an optimization technique for the design of bistable laminates based on an analytical solution for an unsymmetric laminate design. A laminate of arbitrary orthogonal layup with two stable shapes with equal and opposite curvatures was considered by applying the following design rules:

1. Even number of groups of plies, where those below the laminate midplane are rotated 90° with respect to the corresponding ones above the midplane (see Figure 1a); in this work, the number of groups of plies above the midplane (n in Figure 1a) is set to 2;
2. Square edge length L;
3. Each ply thickness about the laminate midplane, $t_{1}$, $t_{2}$, …, $t_{n}$;
4. Each ply is made of the same material.

The stacking sequence, illustrated in Figure 1a, was selected as it provided scope for tailoring the directional stiffness properties, while the orthogonal alignment of plies and the square laminate profile enabled maximum useful deflection between states.

#### 2.3.1. Constrained Optimization Problem

The constrained optimization formulation is introduced as follows:

$$\{\begin{array}{c} \underset{\theta_{1},\theta_{2},t,L}{\min}F\left(x\right):\;\mathrm{Ratio}\;\mathrm{of}\;\mathrm{bending}\;\mathrm{stiffness}\;\mathrm{in}\;\mathrm{two}\;\mathrm{chosen}\\ \mathrm{directions},\;\varphi_{1}\;\mathrm{and}\;\varphi_{2}\\ \mathrm{Subject}\;\mathrm{to}\;\{\begin{array}{c} \mathrm{Bistability}\;\mathrm{constraint}:a+b>0\\ \mathrm{Deflection}\;\mathrm{constraint}:w_{def}=\frac{\left(a_{1}+b_{1}\right)L^{2}}{4} \end{array} \end{array},$$

The optimization problem is solved using MATLAB’s sequential quadratic programming (SQP) method, fmincon. The essential idea of SQP is to model (27) at the current iterate by a quadratic programming subproblem and to use the minimizer of this subproblem to define a new iterate. The keynotes about SQP algorithm is discussed in Appendix B. Furthermore, due to the presence of multiple global and local minima in the optimization domain, MATLAB’s MultiStart algorithm is used to generate potential initial points that are uniformly distributed within the domain in order to identify all minima.

The SMAC structure considered to be fixed at its center when the temperature change from cured ΔT= −160 K (cure 453 K, ambient 293 K) is applied to the structure. In the case of FE simulation, also the out-of-plane displacement was constrained in order to simulate the presence of the mold. 

#### 2.3.2. Objective Function

The formulation of the optimization problem maximizes the bending stiffness in a given direction of loading while at the same time the bending stiffness in the direction of snap to the second stable configuration is minimized (see Figure 1c). The objective function that optimizes the ratio of bending stiffness in two chosen direction of the laminate is represented by Equation (28), where $\varphi_{1}$ is the direction of low bending stiffness, $\varphi_{2}$ is the direction of high bending stiffness and δ is the differential operator,

(28) $$F_{\left(x\right)}=\frac{\delta a_{\varphi_{2}}}{\delta M_{x\varphi_{2}}}/\frac{\delta a_{\varphi_{1}}}{\delta M_{x\varphi_{1}}}.$$

The bending stiffness is characterized as a function of φ by the change of a shape coefficient $a_{\varphi }$ with respect to a moment applied in that direction $M_{x\varphi }$. The definition of the shape coefficients is given in Appendix C. The bending stiffness is evaluated by considering the plate constitutive equations which relate applied forces and moments, N’s and M’s, to the plate curvatures (or shape coefficient), $a_{\varphi }$, $b_{\varphi }$ and $c_{\varphi }$, and midplane strains, $\varepsilon^{0}$’s, using the A, B and D matrices of SMAC and substituting into the plate equation lead to Equation (29). The presence of epoxy/SMA ply is taken in consideration by evaluating the overall ABD matrices of SMAC, i.e., by replacing reduced stiffness terms of composite (${\bar{Q}}_{ij})$ in Equation (22) with ${\bar{Q^{s}}}_{ij}$ terms of reduced stiffness of epoxy/SMA ply at the mid-plane of the composite host laminate. With that, it is acknowledged that the incorporation of SMA wires as an additional layer epoxy/SMA layer at the mid-plane of the composite host laminate violates the above design rules 1 and 4 but, since the contribution of this layer to the overall bending stiffness of the SMAC laminate is negligible (see Section 2.2.2), the approximation related to the violation of the design rules is negligible, too.

Equation (29) is then transformed to align with the direction either $\varphi_{1}$ or $\varphi_{2}$. Note that the subscript φ has been removed from plate curvatures a, b and c in order to avoid confusion in Equation (31). The forces and moments in the transformed direction except $M_{x}$ are set to zero and the remaining system is solved to give an expression in the terms of A, B, D and $M_{x}$. This is used to determine the objective function of Equation (28) which is to be minimized.

(29) $$\left[\begin{array}{c} \begin{array}{c} N_{x}\\ N_{y}\\ N_{xy} \end{array}\\ \begin{array}{c} M_{x}\\ M_{y}\\ M_{xy} \end{array} \end{array}\right]=\left[\begin{array}{cc} \begin{array}{ccc} A_{11} & A_{12} & A_{16}\\ A_{12} & A_{11} & -A_{16}\\ A_{16} & -A_{16} & A_{66} \end{array} & \begin{array}{ccc} B_{11} & 0 & B_{16}\\ 0 & B_{11} & -B_{16}\\ B_{16} & -B_{16} & 0 \end{array}\\ \begin{array}{ccc} B_{11} & 0 & B_{16}\\ 0 & B_{11} & -B_{16}\\ B_{16} & -B_{16} & 0 \end{array} & \begin{array}{ccc} D_{11} & D_{12} & D_{16}\\ D_{12} & D_{11} & -D_{16}\\ D_{16} & -D_{16} & D_{66} \end{array} \end{array}\right]\left[\begin{array}{c} \begin{array}{c} \varepsilon^{0}{}_{x}\\ \varepsilon^{0}{}_{y}\\ \gamma^{0}{}_{xy} \end{array}\\ -a_{\varphi }\\ -b_{\varphi }\\ -c_{\varphi } \end{array}\right],$$

#### 2.3.3. Deflection and Bistability Constraint

The out-of-plane deflection, expressed by Equation (30), is applied as a constraint for the optimization,

(30) $$w\left(x,y\right)=\frac{1}{2}\left(a_{\varphi }x^{2}+b_{\varphi }y^{2}+c_{\varphi }xy\right)\;\kappa_{x}=-\frac{\partial^{2}w}{\partial x^{2}}=-a_{\varphi },\;\kappa_{y}=-\frac{\partial^{2}w}{\partial y^{2}}=-b_{\varphi },\;\kappa_{xy}=-2\frac{\partial^{2}w}{\partial x\partial y}=-c_{\varphi },$$

For a deflection constraint, the out-of-plane displacement at a corner between stable configurations I and II was used (see Figure 1b). The corner deflection between states $w_{def}$ at x=y=L/2 can be expressed by:

(31) $$w_{def}=w_{I}-w_{\mathrm{II}}=\frac{1}{8}\left(a_{\varphi }{}_{I}+b_{\varphi }{}_{I}+c_{\varphi }{}_{I}\right)L^{2}-\frac{1}{8}\left(a_{\varphi }{}_{II}+b_{\varphi }{}_{II}+c_{\varphi }{}_{II}\right)L^{2},$$

The curvatures $a_{\varphi }$, $b_{\varphi }$ and $c_{\varphi }$ can be expressed in terms of the first state (I) alone, as the two states have equal and opposite curvatures. Therefore, the equation had been rewritten in terms of $a_{\varphi }{}_{I}$ and $b_{\varphi }{}_{I}$ only,

(32) $$w_{def}=\frac{1}{4}\left(a_{\varphi }{}_{I}+b_{\varphi }{}_{I}\right)L^{2},$$

A minimum deflection requirement $w_{def}>{\bar{w}}_{def}$ is used to set a restriction on the allowable design space. The inequality was solved as a part of the numerical solution of the optimization problem. It is noted that, as a matter of fact, Equation (32) incorporates the bistability constraint, a+b>0.

## 3. Results and Discussion

### 3.1. Simulation of Uniaxial Behavior of an SMA Wire

The inelastic strain in the SMA wire is the result of the stress-induced martensite phase transformation while it is loaded and unloaded at a temperature below A^0s^. By subsequently heating it above A^0f^, it is possible to recover inelastic strain and return to its parent phase (austenite) by virtue of the reverse transformation. The above phenomenon described as one-way shape memory effect or, simply SME. In this section, SME is exploited to control the shape of a composite plate and to snap its curvature between two stable configurations, therefore a wire of length 300 mm and diameter of 0.1 mm, with material properties listed in Table 2, is first modeled using truss element that can carry only axial loads alike thin, flexible NiTi wires. This in the attempt to emulate the cure cycle of the SMAC and to gain insight about stress distribution, the evolution of martensite volume fraction and transformation strain. The analysis consists of three steps: (i) Pre-strain of the wire at environmental temperature up to 6% elongation; (ii) heating of the wire up to the cure temperature of the composite plate T_cure_ = 453 K, at constant strain; (iii) cooling down to an environmental temperature at constant strain. The results obtained at the end of the first step are also used as an initial condition for SMA wires in the SMAC laminate model as described in Section 3.3. The finite element mesh is composed of 30 linear truss elements of type T3D2 in ABAQUS. The ambient temperature is set to 293 K, which is in between martensite start M^0s^, and austenite starts A^0s^ temperatures (see Table 2). 

Notice that after the cool-down step the strain is kept as if strain recovery was hampered by the presence of the host composite plate.

**Figure 2:**
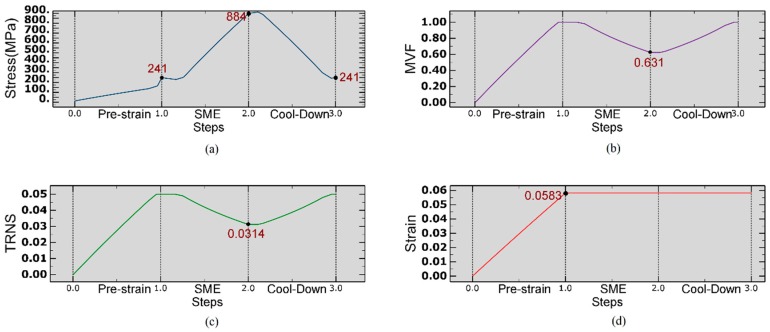
(**a**) Axial stress distribution under mechanical and thermal loads; (**b**) Evolution of Martensite Volume Fraction; (**c**) Induced axial Transformation Strain; (**d**) Total strain induced due to applied displacement.

As shown in Figure 2a, upon heating thermal expansion yields first a drop in axial stress from 241 MPa to 228 MPa, then the axial stress monotonically increases to a value of 884 MPa as the temperature brought up to T_cure_ = 453 K due to the transformation of de-twinned martensite into austenite. This can be observed in Figure 2b,c, as Martensite Volume Fraction (MVF) decreases from ξ = 1 to ξ = 0.631 and transformation strain (TRNS) from $\epsilon^{t}$ = 0.05 to $\epsilon^{t}$ = 0.0314. 

In the cool-down step, the stress initially increases from 884 MPa to 899 MPa due to the thermal contraction of SMA (Figure 2a). As the temperature gradually decreases, high recovery stress which is generated in SMA drops down the stress level which to about 241 MPa at the end of the cooling process. At the same time, MVF gradually increases to unity by accomplishing full phase transformation to de-twinned martensite. It has to be said that, even if the framework chosen to model the constitutive behavior of SMA does not address the transformation of de-twinned into twinned martensite upon cooling, in this case, the martensite remains de-twinned since the final stress level lies above the stress transformation range as shown in Figure 2a, i.e., the martensite is in its fully de-twinned state. 

### 3.2. SMAC Numerical Optimization

The material properties used for SMA and M21/T800 are those shown in Table 2 and Table 3, respectively. The in-plane dimension of the additional SMA ply is equal to the square composite laminate of edge length L, which is a geometric design variable among with the ply orientation θ^1^ and θ^2^ defining the stacking sequence of a four-ply laminate of constant ply thickness t. The SMA ply contains virtually four SMA wires of 0.1 mm diameter and length equal to L. The thickness of the SMA ply is set to $t_{s}$ = 0.2 mm. 

The direction of low bending stiffness $\varphi_{1}$ is set alternatively to 45° or 75° and the direction of high bending stiffness $\varphi_{2}$ is set based on the orientation of SMA wires, that is 0°. The pattern of optimum solutions is detailed in Table 4 and Table 5 for, where the range of design variables and minimum deflection requirement is also shown. The motive behind the chosen set for $w_{def}$ is to draw a clear distinction between the curvature acquired by various SMAC configuration simulated in the Abaqus as shown in Section 3.3.

The two cross-ply solutions ${\left[90^{{}^{\circ}}/90^{{}^{\circ}}/0^{{}^{\circ}}/0^{{}^{\circ}}\right]}_{T}$ and ${\left[-90^{{}^{\circ}}/-90^{{}^{\circ}}/0^{{}^{\circ}}/0^{{}^{\circ}}\right]}_{T}$ for optimization sets A1, B1, C1, D1 and E1 in Table 4, are global optima with the lowest objective function value of 0.4937, 0.4827, 0.4769, 0.4623 and 0.4612 respectively, meaning the chosen direction of high stiffness is approximately two times stiffer than the flexible direction. For a given deflection constraint (sets C1 vs. D1), it has to be noted that the lowest objective function values are similar. This occurs because the effect of a thicker ply found as optimal in D1 is compensated by a shorter edge length in C1. The four sets of design parameters with a higher value of the objective function ($\left[200/-84^{{}^{\circ}}/-53.2^{{}^{\circ}}/0.4\right]$, $\left[277/90^{{}^{\circ}}/-55.7^{{}^{\circ}}/0.41\right]$ and $\left[500/26.04^{{}^{\circ}}/-90^{{}^{\circ}}/0.4\right]$ for sets B1, C1, D1 and E1, respectively), are local optima.

The results in Table 5 are referred to $\varphi_{1}$ = 75° and $\varphi_{2}$ = 0°. The idea behind this example is to understand the dependence on the relative values of the low and high bending stiffness directions. A ${\left[-80^{{}^{\circ}}/-80^{{}^{\circ}}/10^{{}^{\circ}}/10^{{}^{\circ}}\right]}_{T}$ layup is the global optimum for optimization sets A2, B2 and C2 with the lowest objective function value of 0.6983, 0.6921 and 0.6867, respectively, whereas optimization sets D2 and E2 obtain the lowest objective function value for a $\left[-85^{{}^{\circ}}/-26^{{}^{\circ}}/64^{{}^{\circ}}/5^{{}^{\circ}}\right]$ layup. Notice that, for all optimization sets except B2, the last optimal solution in the list shows a small angle between low bending stiffness, $\varphi_{1}$, and ply fiber directions. This results obviously a value of the objective function that is significantly higher, as expected. Therefore, these solutions can be no longer considered global optima.

The minimum deflection requirement and the range of L are gradually increased from a first guess value in order to explore a range of design configurations where the optimal solutions tend to set progressively to the highest value of L and the lowest of t. In this way it is possible to determine a larger set of optimal parameters including local and global optima, that is represented by all the results included in Table 4 and Table 5.

**Figure 3:**
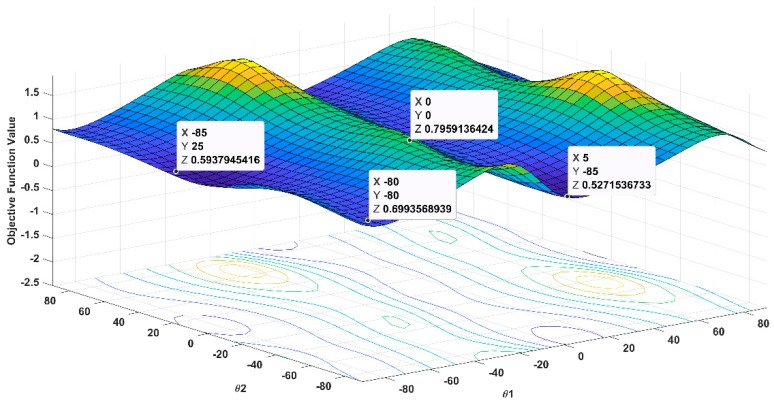
Optimization function as a function of $\theta_{1}$, $\theta_{2}$ for D2 and E2 sets with ply thickness of $t_{1}$ = $t_{2}$ = 0.4 mm.

Figure 3 represents the graphical illustration of the objective function for sets D2 and E2 where the global optimum is located at the point θ_1_ = −85°, θ_2_ = 26° is the correct ply orientation in order to obtain low bending stiffness in $\varphi_{1}$ in comparison with the rotated cross-ply solution ${\left[-{80^{{}^{\circ}}}_{2}/{10^{{}^{\circ}}}_{2}\right]}_{T}$. On the other hand, the rotated cross-ply solution in set D2 and E2 settled down for higher ply thickness as in comparison with the global optima and at these ply orientations, there are many solutions achieving almost equal objective function values. The deflection between states for these solutions vary from 18 to 93 mm. The laminate edge length, which does not affect the objective function, but only the deflection constraint. Therefore, a rotated cross-ply is chosen over the optimal solution with an allowable loss of 12.65% in laminate bending stiffness. Notice that that solution θ_1_ = 5°, θ_2_ = −85° while achieving an objective function value of 0.5272, is discarded since the bi-stability constraint as not satisfied.

### 3.3. FE Simulation of SMAC

In this section, the commercial finite element code ABAQUS is employed to verify the shape after the manufacturing cycle of the SMAC with the optimal design variables determined in the previous section. In particular, the value of *w_def_* of the optimization problem is compared with the one coming from FEA in order to assess the accuracy of the numerical solution. An $L\times L\;{\mathrm{mm}}^{2}$ laminate is modeled in ABAQUS using 4-node, reduced integration shell elements S4R with 4-plies of uniform ply thickness t and a $\left[\theta_{1}/\theta_{2}/90^{{}^{\circ}}+\theta_{1}/90^{{}^{\circ}}+\theta_{2}\right]$ stacking sequence. The embedded SMA wires are modeled using T3D2 truss elements such that the nodes of SMA wires coincide with the nodes of the laminate and a tie constraint is established between the node region of SMA wires and laminate. The tie constraint between the nodes of SMA wires and the laminate physically implies that pre-strained wires are consolidated in the composite matrix and based on this assumption, initial conditions for SMA wire at the beginning of cool-down step are set to the values listed down in Table 6. The Finite Element mesh consists of 100 linear quadrilateral elements of type S4R and 40 linear line elements of type T3D2. 

The out-of-mold shape of the laminate after curing is obtained by cooling down from an initial temperature $T_{cure}$ applied to all the nodes of the model to room temperature. All the nodes of the laminate are constrained in the z-direction while the center node of SMAC is fixed to suppress rigid body motions. At the end of the cooling phase, the constraint in the z-direction is deactivated except at the center node to simulate the opening of the mold. Geometric nonlinearity is accounted for in this step. 

**Figure 4:**
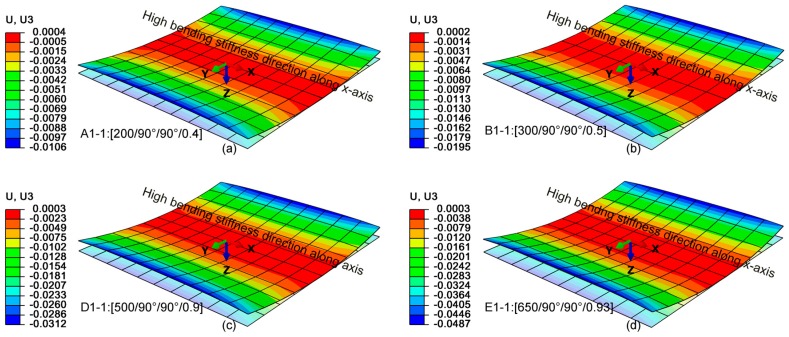
Cured shapes and deflections obtained at the end of the cool-down step (in meters) for sets A1-1, B1-1, D1-1, E1-1 in Table 4.

**Figure 5:**
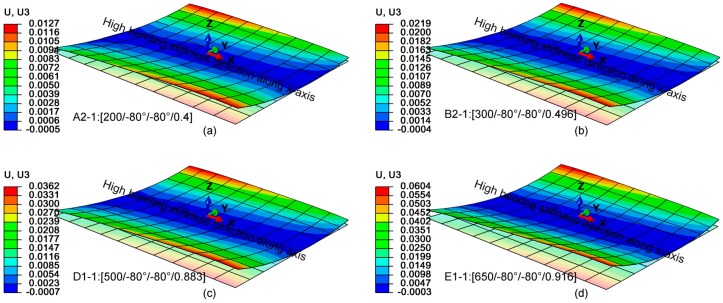
Cured shapes and deflections obtained at the end of the cool-down step (in meters) for sets A2-1, B2-1, D2-2, E2-2 in Table 5.

Figure 4 shows the out-of-plane displacement of the SMAC after the cool-down stage for the optimal sets A1-1, B1-1, D1-1, E1-1 in Table 4, while in Figure 5 it is shown for the sets A2-1, B2-1, D2-2 and E2-2. Even though the latter two are not global optima, they are considered since the values of θ_1_ and θ_2_ are the same of A2-1 and B2-1 cases, therefore facilitating the comparison. It must be underlined that neither L, nor t, nor L/t shows a unique relationship with the deflection, therefore it would have been hard to find the optimal solution for a given deflection simply by trial-and-error. For the results shown in Figure 5, a similar trend can be observed as variations in ply orientations between the laminates are small. Finally, a significant variation in z-displacement can be registered along the y-direction (I stable configuration) in comparison with the x-direction (II stable configuration). Furthermore, a direct correspondence between the attained stable configuration I by SMAC and the objective function criterion to have higher bending stiffness in $\varphi_{2}=0^{{}^{\circ}}$ direction is hard to validate since stable configurations are sensitive to modelled imperfections and uncertainties such as the effect of an additional resin layer which is not modeled in FEA simulations.

**Figure 6:**
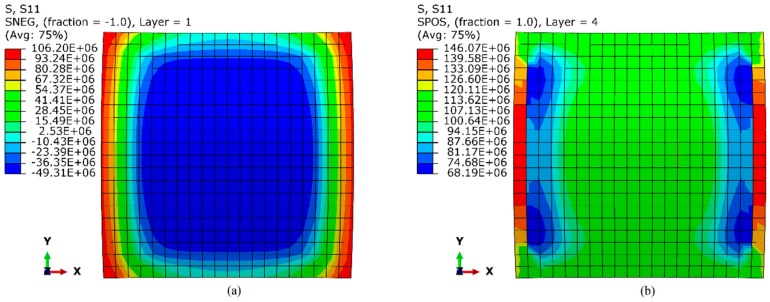
Axial stress distribution in SMAC—(**a**) 0° fiber-oriented bottom layer; (**b**) 90° fiber-oriented top layer.

The results of simulations have been particularly useful in gaining detailed insight into the distribution of longitudinal and transversal stresses in the SMAC after curing. The case of set B1-1- is reported in Figure 6. The 0° (180°)-oriented bottom layer developed compressive stress of −49.31 MPa in fiber direction due to the curvature acquired by the laminate after cool-down step. Note that the peripheral region of the layer is under tension and the inner region under compression, and this state of stress facilitates the snap of the laminate to the second stable configuration [25]. The stresses developed in 90°-oriented top layer is subject to stress concentrations near to the longitudinal edges: the peak tensile stress is +146.4 MPa right along the edges and +68.2 MPa in narrow regions that run longitudinally a small distance into the shell as shown in Figure 6b.

**Figure 7:**
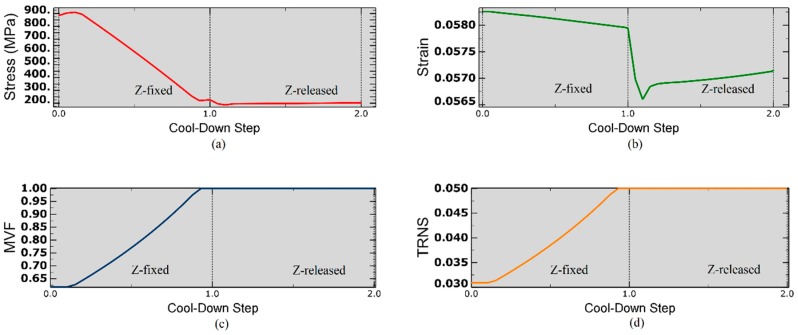
(**a**) Axial stress distribution; (**b**) Induced strain under thermal load; (**c**) Evolution of Martensite Volume Fraction in SMA wires; (**d**) Induced axial Transformation Strain.

Figure 7 depicts the behavior of embedded SMA wires when the initial conditions are set to the values shown in Table 3. In Figure 7a, the stress falls back from 884 MPa to 228 MPa, which is above the stress value requires to transform de-twinned to twinned martensite hence, the initial value of MVF can be preserved for actuation applications. It can be also deduced that the composition of austenite and de-twinned martensite at the beginning of cool-down transforms into full detwinned martensite phase as the final stress level lies above the stress transformation range. Due to cooling, thermal contraction of SMA occurs, and the downward peak, in Figure 7a,b, indicates the release of the constraint in out-of-plane direction (demolding). 

The comparison between the results obtained in ABAQUS and MATLAB is carried out in Table 7 and Table 8 that represents the search space given by $\varphi_{1}$ = 45°, $\varphi_{2}$ = 0° and $\varphi_{1}$ = 75°, $\varphi_{2}$ = 0°, respectively. The resulting difference is contained with 10% and it decreases more sensibly for increasing edge length in comparison to the laminate ply thickness. The incorporation of an SMA ply at the mid-plane in the theoretical model of [26], which is exact in the case of pairs of orthogonal plies, is probably at the basis of the difference between FE and MATLAB results. Since the volume fraction of SMA with respect to whole laminate decreases with the square of edge length and linearly with the total thickness the deviation from the original model due to the presence of SMA ply vanishes for increasing laminate size.

## 4. Conclusions

The efficient modelling technique for the asymmetric, bi-stable composite laminates becomes even more significant when the possibility of actuation by SMA wires is introduced. Therefore, in this work, a design optimization technique for bi-stable composite plates embedded with SMA wires has been developed to find the suitable design variables for a given initial out-of-plane deflection, without having prior knowledge about a “good initial guess”. The optimization problem formulation is based on the minimization of the bending stiffness ratio in two directions, namely $\varphi_{1}$ (low stiffness) and $\varphi_{2}$ (high stiffness) in order to have a plate that is stiff under loading (membrane) and/or bending in $\varphi_{2}$ direction but easy to snap to the other stable configuration by membrane/bending loading in in $\varphi_{1}$ direction. The procedure was implemented in MATLAB the analysis was carried out for $\varphi_{1}$ = 45°, $\varphi_{2}$ = 0° and $\varphi_{1}$ = 75°, $\varphi_{2}$ = 0° low and high stiffness bending direction, respectively, demonstrating the efficiency in finding both global and local optima. Furthermore, the initial deflection $w_{def}$ estimated with MATLAB was compared with a FE analysis done with the software ABAQUS, that yielded a difference within 10%, decreasing down to 1% for increasing laminate size, that confirms the procedure developed in this work as a fast and reliable tool to estimate the optimal design of SMAC bi-stable laminates subjected to an initial deflection constraint. Future work is dedicated to the include in the objective function the SMA wires actuation force necessary to obtain the snap of the laminate to its second stable shape.

## Figures and Tables

**Table 1:** Material properties of M21/T800 prepreg sheet data from [30].

Material Properties	Value
$E_{11}$	157.0 GPa
$E_{22}$	8.5 GPa
$\upsilon_{12}$	0.35
$G_{12}$	4.5 GPa
$\alpha_{1}$	$-0.09\times 10^{-6}{\;K}^{-1}$
$\alpha_{2}$	$30.0\times 10^{-6}{\;K}^{-1}$

**Table 2:** Material parameters for NiTi SMA data from [29].

Material Parameter	Value
Elastic stiffness of the austenite $E^{A}$	70 GPa
Elastic stiffness of the martensite $E^{M}$	30 GPa
Poisson’s ratio (equal for both phases) υ	0.33
Coefficient of thermal expansion for the austenite $\alpha^{A}$	$22.0\times 10^{-6}{\;K}^{-1}$
Coefficient of thermal expansion for the martensite $\alpha^{M}$	$22.0\times 10^{-6}{\;K}^{-1}$
Martensitic start temperature $M^{0s}$	291 K
Martensitic final temperature $M^{0f}$	271 K
Austenitic start temperature $A^{0s}$	295 K
Austenitic final temperature $A^{0f}$	315 K
Maximum transformation strain H	0.05
Stress influence coefficient for austenite $C^{A}$	$7{\;\mathrm{MPa}\;K}^{-1}$
Stress influence coefficient for martensite $C^{M}$	$7{\;\mathrm{MPa}\;K}^{-1}$

**Table 3:** Material properties of Epoxy Resin data from [31].

Mechanical Properties	Value
Tensile Modulus $E_{m}$	10.5 GPa
Flexural Modulus $G_{m}$	10 GPa
Poisson ratio $\upsilon_{m}$	0.475
Coefficient of linear thermal expansion	$34.0\times 10^{-6}{\;K}^{-1}$

**Table 4:** Optimal design parameters for deflection constrained optimization with $\varphi_{1}$ = 45°, $\varphi_{2}$ = 0°.

Optimization Set	Deflection Constraint (mm)	The Range of Geometric Design Variables	Optimal Design Parameters $\left[L/\theta_{1}/\theta_{2}/t\right]$	Objective Function
A1	$w_{def}>18$	L∈[100,200]mm; $\theta_{1},\;\theta_{2}\in \left[-90^{{}^{\circ}},90^{{}^{\circ}}\right];$ t∈[0.4, 2]mm	1 $\left[200/90^{{}^{\circ}}/90^{{}^{\circ}}/0.4\right]$ 2 $\left[200/-84^{{}^{\circ}}/-53.2^{{}^{\circ}}/0.4\right]$	0.4937 0.5752
B1	$w_{def}>35$	L∈[100,300]mm; $\theta_{1},\;\theta_{2}\in \left[-90^{{}^{\circ}},90^{{}^{\circ}}\right];$ t∈[0.4, 2]mm	3 $\left[300/\pm 90^{{}^{\circ}}/\pm 90^{{}^{\circ}}/0.5\right]$ 4 $\left[300/0^{{}^{\circ}}/0^{{}^{\circ}}/0.5\right]$ 5 $\left[277/90^{{}^{\circ}}/-55.7^{{}^{\circ}}/0.41\right]$	0.4827 0.4827 0.6058
C1	$w_{def}>55$	L∈[100,400]mm; $\theta_{1},\;\theta_{2}\in \left[-90^{{}^{\circ}},90^{{}^{\circ}}\right];$ t∈[0.4, 2]mm	1 $\left[400/\pm 90^{{}^{\circ}}/\pm 90^{{}^{\circ}}/0.577\right]$ 2 $\left[400/0^{{}^{\circ}}/0^{{}^{\circ}}/0.577\right]$ 3 $\left[400/\pm 6.36^{{}^{\circ}}/\pm 56.3^{{}^{\circ}}/0.5\right]$	0.4769 0.4769 0.5012
D1	$w_{def}>55$	L∈[100,500]mm; $\theta_{1},\;\theta_{2}\in \left[-90^{{}^{\circ}},90^{{}^{\circ}}\right];$ t∈[0.4, 2]mm	1 $\left[500/\pm 90^{{}^{\circ}}/\pm 90^{{}^{\circ}}/0.9\right]$ 2 $\left[500/0^{{}^{\circ}}/0^{{}^{\circ}}/0.9\right]$ 3 $\left[451/\pm 90^{{}^{\circ}}/\pm 90^{{}^{\circ}}/0.735\right]$ 4 $\left[500/26.4^{{}^{\circ}}/-90^{{}^{\circ}}/0.4\right]$	0.4623 0.4623 0.4682 0.8704
E1	$w_{def}>92$	L∈[100,650]mm; $\theta_{1},\;\theta_{2}\in \left[-90^{{}^{\circ}},90^{{}^{\circ}}\right];$ t∈[0.4, 2]mm	1 $\left[650/\pm 90^{{}^{\circ}}/\pm 90^{{}^{\circ}}/0.93\right]$ 2$\left[650/0^{{}^{\circ}}/0^{{}^{\circ}}/0.93\right]$ 3 $\left[494/\pm 90^{{}^{\circ}}/\pm 90^{{}^{\circ}}/0.532\right]$ 4 $\left[535/-89^{{}^{\circ}}/89^{{}^{\circ}}/0.4015\right]$ 5 $\left[514/-88^{{}^{\circ}}/-81^{{}^{\circ}}/0.4425\right]$	0.4612 0.4612 0.48 0.4935 0.503

**Table 5:** Optimal design parameters for deflection constrained optimization with $\varphi_{1}$ = 75°, $\varphi_{2}$ = 0°.

Optimization Set	Deflection Constraint (mm)	The Range of Geometric Design Variables	Optimal Design Parameters $\left[L/\theta_{1}/\theta_{2}/t\right]$	Objective Function
A2	$w_{def}>18$	L∈[100,200]mm; $\theta_{1},\;\theta_{2}\in \left[-90^{{}^{\circ}},90^{{}^{\circ}}\right];$ t∈[0.4, 2]mm	1 $\left[200/-80^{{}^{\circ}}/-80^{{}^{\circ}}/0.4\right]$ 2 $\left[200/90^{{}^{\circ}}/54.1^{{}^{\circ}}/0.4\right]$	0.6994 0.7628
B2	$w_{def}>35$	L∈[100,300]mm; $\theta_{1},\;\theta_{2}\in \left[-90^{{}^{\circ}},90^{{}^{\circ}}\right];$ t∈[0.4, 2]mm	1 $\left[300/-80^{{}^{\circ}}/-80^{{}^{\circ}}/0.496\right]$ 2 $\left[300/10^{{}^{\circ}}/10^{{}^{\circ}}/0.496\right]$ 3 $\left[300/-82^{{}^{\circ}}/90^{{}^{\circ}}/0.52\right]$	0.6921 0.6921 0.7044
C2	$w_{def}>55$	L∈[100,400]mm; $\theta_{1},\;\theta_{2}\in \left[-90^{{}^{\circ}},90^{{}^{\circ}}\right];$ t∈[0.4, 2]mm	1 $\left[400/-80^{{}^{\circ}}/-80^{{}^{\circ}}/0.571\right]$ 2 $\left[400/10^{{}^{\circ}}/10^{{}^{\circ}}/0.571\right]$ 3 $\left[400/18.1^{{}^{\circ}}/65.2^{{}^{\circ}}/0.4\right]$ 4 $\left[400/\pm 90^{{}^{\circ}}/\pm 90^{{}^{\circ}}/0.5\right]$	0.6867 0.6867 0.7173 0.7888
D2	$w_{def}>55$	L∈[100,500]mm; $\theta_{1},\;\theta_{2}\in \left[-90^{{}^{\circ}},90^{{}^{\circ}}\right];$ t∈[0.4, 2]mm	1 $\left[500/-85^{{}^{\circ}}/26^{{}^{\circ}}/0.4\right]$ 2 $\left[500/-80^{{}^{\circ}}/-80^{{}^{\circ}}/0.883\right]$ 3 $\left[500/10^{{}^{\circ}}/10^{{}^{\circ}}/0.883\right]$4 $\left[500/\pm 90^{{}^{\circ}}/\pm 90^{{}^{\circ}}/0.82\right]$	0.5996 0.6755 0.6755 0.7765
E2	$w_{def}>92$	L∈[100,650]mm; $\theta_{1},\;\theta_{2}\in \left[-90^{{}^{\circ}},90^{{}^{\circ}}\right];$ t∈[0.4, 2]mm	1 $\left[500/-85^{{}^{\circ}}/26^{{}^{\circ}}/0.4\right]$ 2 $\left[650/-80^{{}^{\circ}}/-80^{{}^{\circ}}/0.916\right]$ 3 $\left[650/10^{{}^{\circ}}/10^{{}^{\circ}}/0.916\right]$ 4 $\left[595/13.3^{{}^{\circ}}/25.5^{{}^{\circ}}/0.487\right]$ 5 $\left[650/\pm 90^{{}^{\circ}}/\pm 90^{{}^{\circ}}/0.9241\right]$	0.5977 0.6747 0.6747 0.7167 0.7741

**Table 6:** SMA wires initial condition at cool-down step.

Axial Stress $\sigma_{11}^{s}$	Total Strain $\varepsilon_{11}^{s}$	MVF ξ	TRNS $\epsilon_{11}^{t}$
884 MPa	0.0583	0.631	0.0314

**Table 7:** Comparison of $w_{def}$ obtained from ABAQUS’s model based on the optimal design parameters for the search space given by $\varphi_{1}$ = 45°, $\varphi_{2}$ = 0°.

Optimization Set	$w_{def}$ in ABAQUS (mm)	$w_{def}$ in MATLAB (mm)	Percentage Difference (%)
A1-1	21.19	18.9	10.8
B1-1	39.05	37.748	3.33
C1-1	59.57	58.722	1.42
D1-1	59.08	58.6	0.813
E1-1	97.31	98.15	0.863

**Table 8:** Comparison of $w_{def}$ obtained from ABAQUS’s model based on the optimal design parameters for the search space given by $\varphi_{1}$ = 75°, $\varphi_{2}$ = 0°.

Optimization Set	$w_{def}$ in ABAQUS (mm)	$w_{def}$ in MATLAB (mm)	Percentage Difference (%)
A2-1	20.53	18.5	9.88
B2-1	37.86	35.6	5.79
C2-1	61.58	59.14	3.96
D2-2	62.6	63.5	1.44
E2-2	102.76	101.91	0.827

**Figure A1:**
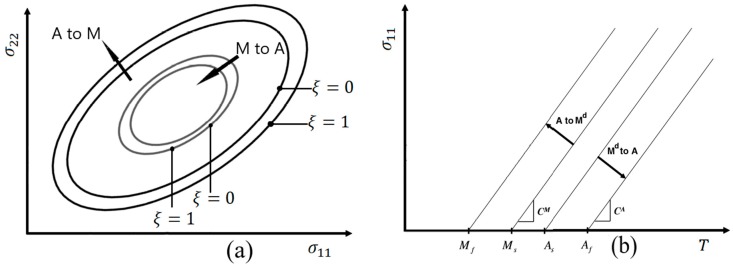
(**a**) Transformation surface plot in a two-dimensional stress space for forward and reverse phase transformation at a given fixed temperature (T = 308 K); (**b**) schematics of stress-temperature phase diagram for an SMA.

## Appendix A

The stress and temperature conditions for phase transformation involves the determination of transformation “surfaces,” or boundaries of the transformation regions in a stress-temperature space. These indicate where a given transformation begins or ends. These four surfaces, and their equations (given below) point out the following, from first to last: The beginning of forward phase transformation; the end of forward phase transformation; the beginning of reverse phase transformation and end of reverse phase transformation, respectively, at zero stress. For a stress-space $\left(\sigma_{11},\;\sigma_{22}\right)$, the three-dimensional plot of the transformation surfaces for forward or reverse phase transformation can be presented in the σ-T space. In Figure A1a, a slice of the transformation surface boundaries of a 3D plot at a constant temperature is shown.

$$\{\begin{array}{ccccc} \pi \left(\sigma ,T,\xi \right)=Y & \mathrm{at} & \sigma =0, & T=M_{s}, & \xi =0.\\ \pi \left(\sigma ,T,\xi \right)=Y & \mathrm{at} & \sigma =0, & T=M_{f}, & \xi =1.\\ \pi \left(\sigma ,T,\xi \right)=-Y & \mathrm{at} & \sigma =0, & T=A_{s}, & \xi =1.\\ \pi \left(\sigma ,T,\xi \right)=-Y & \mathrm{at} & \sigma =0, & T=A_{f}, & \xi =0. \end{array}$$

Similarly, considering $\sigma_{22}=0$ the boundaries of the transformation surfaces in the uniaxial σ-T space is shown in Figure A1b. The form of these boundaries is assumed to be linear (it might be quadratic which depend on the behavior of material) and irrespective of their form, these boundaries are partially described by their intersections with the zero-stress axis and by the slopes at some stress level (e.g., zero stress). Such slopes are known as “stress influence coefficients” [32].

The value of entropy changes in the forward or reverse transformation (exothermic and endothermic processes) can be calculated through a Clausius-Clapeyron-like relation [33]:

$$\Delta S=-\frac{d\sigma }{dT}\epsilon^{t};\;\frac{d\sigma }{dT}=C^{A}=C^{M},$$

where, ΔS is a change in the entropy, $\epsilon^{t}$ is transformation strain, $C^{A}$ and $C^{M}$ represent stress influence coefficient for austenite and martensite. The entropy relation holds for forward transformation (A→M) by accounting for a decrease in entropy as the process is exothermic in nature however, it violates Le Chatelier-Braun principle for reverse transformation (M→A). This is because $\epsilon^{t}$ does not provide any information about the reversal of the phase transformation hence, it failed to evaluate the increase in entropy during the endothermic process (M→A). This additional information about the phase reversal can be understood by observing the rate of transformation at each time increment therefore, the above relation is modified as follows:

$$\dot{\Delta S}=-\frac{d\sigma }{dT}\dot{\epsilon^{t}};\;\begin{array}{cc} \dot{\epsilon^{t}}>0 & A\to M\\ \dot{\epsilon^{t}}<0 & M\to A \end{array},$$

Hence, a Clausius-Clapeyron-like relation justifies Le Chatelier-Braun principle for both forward and reverse transformation.

## Appendix B

### Appendix B.1. Karush-Kuhn-Tucker (KKT) Conditions and the Lagrangian Function

The general Nonlinear Programming (NLP) problem

$$\{\begin{array}{cc} \min F\left(x\right) & \\ s.t.h_{i}\left(x\right)=0 & i\in E\\ \mathrm{and}\;g_{i}\left(x\right)\geq 0 & i\in I \end{array}\;\left(\mathrm{active}\right).$$

The Lagrangian function formulates the problem into one function using Lagrangian multipliers λ for equality constraints and μ for inequality constraints:

$$L\left(x,\lambda ,\mu \right)=F\left(x\right)+\underset{i}{\sum }\lambda_{i}h_{i}\left(x\right)+\underset{i}{\sum }\mu_{i}g_{i}\left(x\right).$$

A single function can be optimized by finding critical points where the gradient is zero. This procedure now includes λ and μ as variables. The system formed from this gradient yields the KKT conditions:

$$\nabla L=\left[\begin{array}{c} \frac{dL}{dx}\\ \frac{dL}{d\lambda }\\ \frac{dy}{d\mu } \end{array}\right]=\left[\begin{array}{c} \nabla F+\lambda \nabla h+\mu \nabla g^{*}\\ h\\ g^{*} \end{array}\right]=0.$$

### Appendix B.2. The SQP Algorithm

Critical points of the objective function will also be critical points of the Lagrangian function and vice versa because the Lagrangian function is equal to the objective function at a KKT point; all constraints are either equal to zero or inactive. The algorithm is thus simply iterating Newton’s method to find critical points of the Lagrangian function. Since the Lagrangian multipliers are additional variables, the iteration forms a system:

$$\left[\begin{array}{c} x_{k+1}\\ \lambda_{k+1}\\ \mu_{k+1} \end{array}\right]=\left[\begin{array}{c} x_{k}\\ \lambda_{k}\\ \mu_{k} \end{array}\right]-{\left(\nabla^{2}L_{k}\right)}^{-1}\nabla L_{k}=\left[\begin{array}{c} x_{k}\\ \lambda_{k}\\ \mu_{k} \end{array}\right]-p,$$

The improvement direction p for Newton’s method iterations is found with a quadratic minimization sub-problem that is solved using quadratic algorithms.

$$\begin{array}{c} \underset{p}{\min}F_{k}\left(x\right)+\nabla f_{k}^{T}p+\frac{1}{2}p^{T}\nabla_{xx}^{2}L_{k}p\\ s.t\;\nabla h_{k}p+h_{k}=0\\ and\;\nabla g_{k}p+g_{k}=0. \end{array}$$

This problem is quadratic and thus must be solved with non-linear methods, which once again introduces the need to solve a non-linear problem into the algorithm, but this predictable sub-problem with one variable is much easier to tackle than the parent problem. This subproblem can be solved using any Quadratic Programming algorithm.

## Appendix C

A Rayleigh-Ritz method of minimisation of the total strain energy of the laminate is used to obtain the shape coeffiecents of the laminate’s shapes. The total strain energy of the laminate W can then be expressed as

$$W=\int_{\frac{-L_{x}}{2}}^{\frac{L_{x}}{2}}\int_{\frac{-L_{y}}{2}}^{\frac{L_{y}}{2}}\int_{\frac{H}{2}}^{\frac{H}{2}}(\frac{1}{2}{\bar{Q}}_{11}\varepsilon_{x}^{2}+{\bar{Q}}_{12}\varepsilon_{x}\varepsilon_{y}+{\bar{Q}}_{16}\varepsilon_{xy}\varepsilon_{x}+\frac{1}{2}{\bar{Q}}_{22}\varepsilon_{y}^{2}+{\bar{Q}}_{26}\varepsilon_{xy}\varepsilon_{y}+\frac{1}{2}{\bar{Q}}_{66}\varepsilon_{xy}^{2}\;-\left({\bar{Q}}_{11}\alpha_{x}+{\bar{Q}}_{12}\alpha_{y}+{\bar{Q}}_{16}\alpha_{xy}\right)\varepsilon_{x}\Delta T-\left({\bar{Q}}_{12}\alpha_{x}+{\bar{Q}}_{22}\alpha_{y}+{\bar{Q}}_{26}\alpha_{xy}\right)\varepsilon_{y}\Delta T-({\bar{Q}}_{16}\alpha_{x}\;+{\bar{Q}}_{26}\alpha_{y}+{\bar{Q}}_{66}\alpha_{xy})\varepsilon_{xy}\Delta T)dxdydz$$

where the ${\bar{Q}}_{ij}$’s are the symmetric transformed stiffness matrices of the individual layers, $L_{x}$ and $L_{y}$ are the side lengths of the laminate, H is the total laminate thickness and ε’s are the total strains as stated in Equation (13). Classical laminate theory follows the assumption that through-thickness stresses are small in comparison to in-plane stresses. In assuming that, the normal stress in the z-direction and out-of-plane shear stresses are assumed to be zero, and leads to an expression for the total energy as a function of material and geometric properties, the temperature change and the total strains.

The midplane strain in Equation (12) are approximated by third order polynomial. Based on, Dano and Hyer [34] findings the form of the midplane strains can be reduced as follows:

$$\begin{array}{c} \varepsilon^{0}{}_{x}=d_{1}+d_{1}x^{2}+d_{3}xy+d_{4}y^{2}\\ \varepsilon^{0}{}_{y}=d_{5}+d_{6}x^{2}+d_{7}xy+d_{8}y^{2}\\ \varepsilon^{0}{}_{xy}=2d_{9}+(ab-\frac{c^{2}}{4}+2d_{4}+2d_{6})x^{2}+\left(\frac{1}{2}\left(\frac{ac}{2}+d_{3}\right)+d_{10}\right)xy+\left(\frac{1}{2}\left(\frac{bc}{2}+d_{7}\right)+d_{11}\right)y^{2} \end{array},$$

The spatial integrations results in an expression for the total strain energy of the lamintae as follows:

$$W=W\left(a,b,c,d_{1},d_{2},d_{3},d_{4},d_{5},d_{6},d_{7},d_{8},d_{9},d_{10},d_{11}\right),$$

To find the minimum energy state it is required that

$$\delta W=\sum_{i=1}^{14}\frac{\partial W}{\partial p_{i}}\partial p_{i}=0,$$

where, $p_{i}$’s are $a,b,c,d_{1},\;\ldots ,d_{11}$. To meet this requirement it is necessary to have

$$L_{i}=\frac{\partial W}{\partial p_{i}}=0;i=1,2,\;\ldots ,\;14,$$

This results in 14 nonlinear equilibrium equations to be solved to find the stable shapes defined by the 14 shape coefficients $p_{i}$. The solutions of the equilibrium equations give the configurations of the laminate at the room temperature. Based on the design rules (Section 2.3), it possible to reduce the above system to just 3 equations and 3 unknows a, b and c and further details about the analytical solution is presented in [26].

## References

[1] Kudva J.N. (2004). Overview of the DARPA smart wing project. JIMSS.

[2] Lachenal X., Daynes S., Weaver P.M. (2013). Review of morphing concepts and material for wind turbine blade application. Wind Energy.

[3] Hyer M.W. (1981). Calculations of the room-temperature shapes of unsymmetric laminates. J. Comp. Mater..

[4] Hyer M.W. (1981). Some observations on the cured shape of thin unsymmetric laminates. J. Comp. Mater..

[5] Jun W.J., Hong C.S. (1990). Effect of Residual Shear Strain on the Cured Shape of Unsymmetric Cross-ply Thin Laminates. J. Comp. Mater..

[6] Schlecht M., Schulte K. (1999). Advanced Calculations of the Room-temperature Shapes of Unsymmetric Laminates. J. Comp. Mater..

[7] Tawfik S., Tan X., Ozbay S., Armanios E. (1999). Anticlastic Stability Modeling for Cross-ply Composites. J. Comp. Mater..

[8] Bo Z., Lagoudas D. (1999). Thermomechanical modelling of polycrystalline SMAs under cyclic loading, Part III: Evolution of plastic strains and two-way shape memory effect. IJES.

[9] Boyd J., Lagoudas D. (1996). A thermodynamical constitutive model for shape memory materials. Part I: The monolithic shape memory alloy. Int. J. Plast..

[10] Brinson L. (1993). One-dimensional constitutive behavior of shape memory alloys: thermomechanical derivation with non-constant material functions and redefined martensite internal variable. JIMSS.

[11] Liang C., Rogers C. (1990). One-dimensional thermomechanical constitutive relations for shape memory materials. JIMSS.

[12] Qidwai M., Lagoudas D. (2000). On thermomechanics and transformation surfaces of polycrystalline NiTi shape memory alloy material. Int. J. Plast..

[13] Qidwai M., Lagoudas D. (2000). Numerical implementation of a shape memory alloy thermomechanical constitutive model using return mapping algorithms. IJNME.

[14] Tanaka K., Kobayashi S., Sato Y. (1986). Thermomechanics of transformation pseudoelasticity and shape memory effect in alloys. Int. J. Plast..

[15] Duerig T.W., Melton K.N., Stoeckel D., Wayman C.M. (1990). Engineering Aspects of Shape Memory Alloys.

[16] Stachiv I., Sittner P., Olejnicek J., Landa M., Heller L. (2017). Exploiting NiTi shape memory alloy films in design of tunable high frequency microcantilever resonators. Appl. Phys. Lett..

[17] Stachiv I., Sittner P. (2018). Nanocantilevers with Adjustable Static Deflection and Significantly Tunable Spectrum Resonant Frequencies for Applications in Nanomechanical Mass Sensors. Nanomaterials.

[18] Ryu J., Lee J., Cho M., Kim S.-W., Koh J.-S., Cho K.-J. Snap-through behavior of bi-stable composite structure using SMA spring actuator. Proceedings of the 52nd AIAA/ASME/ASCE/AHS/ASC Structures, Structural Dynamics, and Material Conference.

[19] Dano M.-L., Hyer M.W. (2003). SMA-induced snap-through of unsymmetric fiber-reinforced composite laminates. Int. J. Solids Struct..

[20] Hassanli S., Samali B. (2016). Buckling analysis of laminated composite curved panels reinforced with linear and non-linear distribution of Shape Memory Alloys. Thin-Walled Struct..

[21] Tawfik M., Ro J.J., Mei C. (2002). Thermal post-buckling and aeroelastic behaviour of shape memory alloy reinforced plates. Smart Mater. Struct..

[22] Turner T.L. (2000). A new thermoelastic model for analysis of shape memory alloy hybrid composites. J. Intell. Mater. Syst. Struct..

[23] Niknami A., Shariyat M. (2017). Influence of the heat generation on the phase transformations and impact responses of composite plates with embedded SMA wires. J. Comput. Appl. Res. Mech. Eng..

[24] Birman V., Chandrashekhara K., Sain S. (1996). An approach to optimization of shape memory alloy hybrid composite plates subjected to low-velocity impact. Composites Part B Eng..

[25] Gandhi Y., Pirondi A., Collini L. Analysis of bistable composite laminate with embedded SMA actuators. Proceedings of the 47th AIAS International Conference on Stress Analysis.

[26] Betts D.N. (2012). Modelling and Optimization of Bistable Composite Laminates. Ph.D. Thesis.

[27] Nocedal J., Wright S. (1999). Numerical Optimization.

[28] Sequential Quadratic Programming. https://optimization.mccormick.northwestern.edu/index.php/Sequential_quadratic_programming.

[29] Lagoudas D., Bo Z., Qidwai M., Entchev P. (2003). SMA_UM: User Material Subroutine for Thermomechanical Constitutive Model of Shape Memory Alloys.

[30] Hexcel. https://www.hexcel.com/Resources/DataSheets/.

[31] Simmons. http://www.epoxyworktops.com/epoxy-resin/mech-properties.html.

[32] Hartl D.J., Lagoudas D.C., Lagoudas D.C. (2008). Thermomechanical Characterization of Shape Memory Alloy Materials. Shape Memory Alloy: Modeling and Engineering Applications.

[33] Liu Y., McCormick P.G. (1994). Thermodynamic analysis of the martensitic transformation in NiTi-II. Effect of transformation cycling. Acta Metall. Mater..

[34] Dano M.L., Hyer M.W. (1988). Thermally-induced deformation behaviour of unsymmetric laminates. Int. J. Solids Struct..

